# Age, empathy, familiarity, domestication and call features enhance human perception of animal emotion expressions

**DOI:** 10.1098/rsos.221138

**Published:** 2022-12-07

**Authors:** Jasmin Sowerby Greenall, Lydia Cornu, Anne-Laure Maigrot, Monica Padilla de la Torre, Elodie F. Briefer

**Affiliations:** ^1^ Institute of Agricultural Sciences, ETH Zürich, Universitätsstrasse 2, 8092 Zurich, Switzerland; ^2^ Behavioural Ecology Group, Section for Ecology & Evolution, Department of Biology, University of Copenhagen, 2100 Copenhagen Ø, Denmark; ^3^ Swiss National Stud Farm, Agroscope, Les Longs-Prés, 1580 Avenches, Switzerland; ^4^ Wildlife Ecology & Conservation Group, Wageningen University and Research, 6708PB Wageningen, The Netherlands

**Keywords:** arousal, cross-species, emotions, ungulates, valence, vocalizations

## Abstract

Vocalizations constitute an effective way to communicate both emotional arousal (bodily activation) and valence (negative/positive). There is strong evidence suggesting that the convergence of vocal expression of emotional arousal among animal species occurs, hence enabling cross-species perception of arousal, but it is not clear if the same is true for emotional valence. Here, we conducted a large online survey to test the ability of humans to perceive emotions in the contact calls of several wild and domestic ungulates produced in situations of known emotional arousal (previously validated using either heart rate or locomotion) and valence (validated based on the context of production and behavioural indicators of emotions). Participants (1024 respondents from 48 countries) were able to rate above chance levels the arousal level of vocalizations of three of the six ungulate species and the valence of four of them. Percentages of correct ratings did not differ a lot across species for arousal (49–59%), while they showed much more variation for valence (33–68%). Interestingly, several factors such as age, empathy, familiarity and specific features of the calls enhanced these scores. These findings suggest the existence of a shared emotional system across mammalian species, which is much more pronounced for arousal than valence.

## 1. Introduction

Back in the nineteenth century, Darwin described similarities between how humans and other animals express emotions [1], and suggested that human vocal expression of emotions could date back to our earliest terrestrial ancestors [2]. Emotions are intense, short-term reactions triggered in response to specific internal or external stimuli, characterized by a certain arousal level (bodily activation) and valence (positive versus negative) [3]. These reactions are composed of coordinated behavioural, neurophysiological and cognitive changes, which can be measured both in humans and in non-human animals, and a subjective, conscious perception of these changes (feeling) quantifiable in humans [4]. As part of behavioural changes, both humans and other animals often vocalize. The structure (duration, frequencies and amplitude) of these vocalizations is influenced notably by emotion-related neurophysiological changes, and thus conveys indicators of experienced emotions and serves to regulate social interaction within groups [5,6].

Research on emotions in non-human animals has provided increasing evidence that physiological parameters are mainly affected by emotional arousal, rather than valence (e.g. [7]). Cross-species acoustic universals in the expression of emotional arousal are predicted to exist [8], given that vocal production mechanisms and the physiological basis of emotions (e.g. stress pathways) are highly conserved among vertebrates. Accordingly, vocalizations produced under high arousal are characterized by higher amplitudes, higher rates of production, higher frequencies and a more variable fundamental frequency (*f0*, lowest frequency of a periodic wave) across vertebrates [9,10]. Vocal expression of emotional valence, by contrast, could be more species specific, but a trend can be observed across species, with vocalizations associated with positive valence being shorter, with a lower and less variable *f0* [5,9]. The question hence remains whether Darwin's prediction is valid also for vocal expression of emotional valence.

To ascertain whether the expression of emotions has been conserved throughout evolution, we can test if cross-cultural and/or cross-species perception of emotion exists (i.e. the ability to decipher emotions in the vocalizations of other cultures and/or other species). Humans are able to accurately infer vocal expression across languages and cultures [11]. They are also able to decipher the arousal associated with the vocalizations of several species, ranging from frogs to bonobos [10,12]. By contrast, the ability of humans to rate the valence of animal vocalizations seems to vary between species and studies [13–16]. In the majority of studies investigating valence recognition, people were asked to judge the emotional valence associated with different types of calls (e.g. pig grunts versus screams; human cries versus laughs; but see [15,17–19] for exceptions). The strikingly different structure of these sounds makes them easier to recognize and categorize. In addition, a major limitation of the studies published so far is that, to our knowledge, the arousal and valence of the producers were always inferred from the context of production only, and not validated using emotional indicators. More research is thus needed to test if humans can perceive subtle differences in animal calls conveying information about emotional arousal and valence.

**Figure 1.**
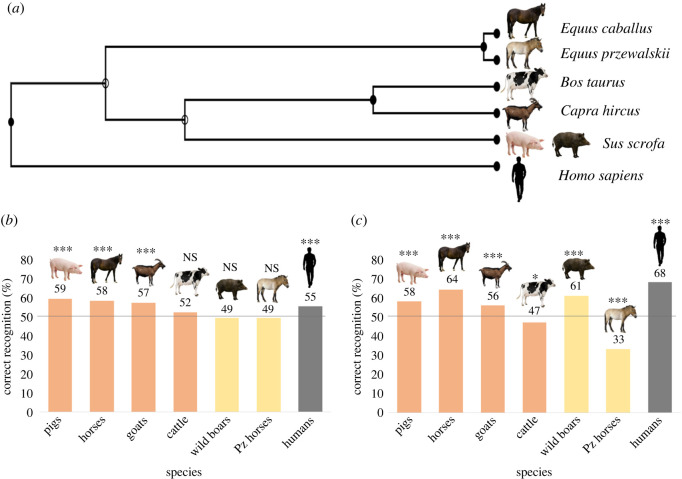
(*a*) Phylogeny of the species played back in the survey. Correct recognition percentage per species for (*b*) arousal and (*c*) valence questions (orange: domestic species; yellow: wild species; grey: humans; binomial test: *0.05 ≤ *p* < 0.01, ***p ≤ 0.0001, NS = not significant).

In the present study, we investigated if humans are able to perceive subtle changes in the acoustic structure of the vocalizations of domestic ungulates (horses, *Equus caballus*; pigs, *Sus scrofa domesticus*; goats, *Capra hircus*; and cattle, *Bos taurus*) and their closely related species (Przewalski's horses, *Equus przewalskii*; and wild boars, *Sus scrofa*; figure 1*a*), as a function of either the emotional arousal or valence of the caller. To this aim, we conducted an online survey using vocalizations produced in situations of known emotional arousal (validated using either heart rate or locomotion) and valence (validated based on the context of production and behavioural indicators) [7,20–24], which was translated into eight languages. Cross-species recognition of emotions or of the context of vocal production seems to be affected by several factors, such as demographic factors (e.g. gender [25] and age [26]), familiarity with the species (experience-driven cognitive processes [17,27,28]) or the acoustic parameters of the calls played [10,12]. Other factors, such as phylogeny (evolutionarily retained mechanisms), whose effect has been tested directly [16] or indirectly by playing the sounds of several species [10,12,14], domestication, whose effect remains yet untested, as well as empathy (i.e. ‘the capacity to be affected by and share the emotional state of another’ [29]), whose effect has been poorly investigated, could also affect human perception of animal emotions. We thus also investigated the factors affecting cross-species perception of emotions.

First, we investigated the effect of the emotional dimension. Vocal expression of emotional arousal seems well conserved throughout evolution, while the expression of emotional valence could be more species specific [5,9]. Expressions of humans and other animals are thus expected to be more similar for arousal than for valence. As a result, cross-species recognition of emotional arousal could be easier than recognition of emotional valence. Accordingly, we expected that participants' correct ratings (their ‘scores’) would be higher for arousal than valence.

Second, we investigated the effect of demographical factors (age, gender and education). As empathy is thought to have evolved from the mother–offspring bond [30,31], people with children (and particularly women) are expected to be better at recognizing emotions of others. Accordingly, women are known to have a higher emotional sensitivity [32], and have been shown to rate the context of production of dog growls [28] and pig vocalizations [33] more accurately than men. In children, the identification of vocal expression of emotions develops gradually with age [34], while decreased performances at older ages have also been shown for certain emotions across modalities [35]. In line with these findings, an effect of participants' age has been shown in recognizing the emotions of dogs [26] and stump-tailed macaques [36]. In addition, education could affect people's abilities to perceive the emotions of other species, as this factor might have an impact on emotional competence as a result of higher cognitive capacity or intelligence [37]. Based on the literature, we thus expected women, participants in the middle age range, those with children and those with a higher education to obtain higher scores.

Third, we investigated the effect of empathy towards other humans. Perception of the emotional state of others is the basis of empathy [29]. In line with this statement, empathy has been shown to affect recognition of facial expression of emotions in both humans and dogs [38]. High emotional contagion and empathy have also recently been shown to enhance our ability to detect emotional authenticity in human laughter [39]. We thus predicted that the more participants are assessed as empathic, the higher their likelihood of recognizing emotions of animal vocalizations (i.e. their scores) will be.

Fourth, we investigated the effect of familiarity with the species. Empathy, and hence emotion recognition, is predicted to be higher among familiar individuals [31]. Accordingly, although cross-language and cross-culture recognition of vocal expression of emotions does occur, it is higher within one's own culture and mother tongue [11], suggesting a role of familiarity in emotion recognition. An increase in emotion recognition, or in correct assignment of production contexts, as a function of familiarity with the species or the emitter has been found when participants were asked to judge vocalizations of cats [17–19,40], pigs [27], chimpanzees and tree shrews [16], while mixed results have been found in dogs [16,26,28,41]. Therefore, we also expected that participants that are more familiar with animals in general, and with the species used in our survey, would obtain higher scores.

Fifth, we investigated the effect of domestication. Domestication constitutes another factor that could have strongly influenced human–animal communication [42]. Throughout the thousands of years that domestic animals have been co-evolving with humans, humans might have developed an ability to assess animal vocalizations, or the animals might have evolved to influence human behaviour more efficiently [33]. Alternatively, domestic animals may have been selected according to their similarity of emotion expression with humans, as this would enhance human–animal communication. We thus predicted that participants would obtain higher scores when rating domestic compared to wild species. To our knowledge, this is the first study comparing how people judge emotion expressions of domestic animals and their wild counterparts (but see [43] testing human perception of selected lines of silver foxes).

Lastly, we investigated the effect of the acoustic parameters of the sounds played. Specific parameters of the vocalizations (e.g. fundamental frequency and spectral centre of gravity) have been shown to affect how people rate animal calls, as they tend to rely on the same basic rules that are valid for rating conspecific vocalizations [10,12,25,41]. Since participants in our study were always presented with a choice of two vocalizations to compare, we hypothesized that (i) scores would improve when the difference in certain vocal parameters of importance between these two sounds was larger, as it would make the sounds more distinct and hence easier to categorize as low or high arousal, or as negative or positive; (ii) the perception of the vocalizations by the participants (independently of whether they were correct or not) as low or high arousal, or as negative or positive, would depend on certain parameters of importance. We predicted that the parameters facilitating arousal recognition would be those commonly associated with this dimension (e.g. spectral centre of gravity and *f0*), while the parameters facilitating valence recognition would be those shown to vary between negative and positive contexts of production across species (i.e. sound duration and *f0*) [5,9]. We also predicted that the parameters facilitating arousal recognition would be similar across species, while those enabling valence recognition would differ between species.

## 2. Material and methods

### 2.1. Participants

Data were collected through an online survey built in Qualtrics, live from 12 October 2016 to 3 January 2017. Participants were recruited by means of an advert, which briefly stated the purpose of the study and was circulated by email, through social media, and through a magazine and TV show. In total, 1024 participants finished the survey (*M* = 262, *F* = 747, prefer not to say = 15, from 48 countries of origin; electronic supplementary material, table S1). Participation was voluntary, anonymous and not remunerated.

### 2.2. Survey procedure

The survey was available in English, French, Spanish, Italian, Portuguese, German, Dutch and Hebrew. The original version was built in English and subsequently translated into other languages by one native speaker and verified (back translated) by another native or bilingual speaker. Before launching the survey, it was tested by eight participants and refined. The final version took approximately 10 min to complete. It was made up of five main parts: an introduction and consent to participate, a set of demographic questions and questions on experience with each species, an introduction to the concepts of emotional arousal and valence along with two trial questions, a set of questions with sounds to rate, and finally an empathy questionnaire.

#### 2.2.1. Introduction and consent

First, participants were asked to give their consent to participate and were informed that they could stop the survey at any time, and that their data would be stored anonymously and handled confidentially. They were then asked to confirm that they had no hearing problems that they knew of and that they were completing the survey on a computer with external speakers or headphones. They were also asked to play a test sound to check that they were able to hear it clearly, and to adjust the volume before beginning.

#### 2.2.2. Demography and experience with each species

Participants then completed a set of demographic questions (gender, age, if they had children or not, country of origin, country of residence and education). For gender and age, they had the opportunity to answer ‘I prefer not to say’. They were then asked if their work and studies were related to animals (yes or no), and about their level of experience with each of the species; i.e. how often they were in contact with each species included in the survey (answers ranging from ‘never’ to ‘daily’).

#### 2.2.3. Concepts and trial questions

Before rating the sounds, participants read an explanation of the aim of the study and the concept of emotional arousal and valence, and completed two training questions (one for valence and one for arousal), to ensure their understanding of the format. They also had the option to redo this section before moving on to the sound ratings.

#### 2.2.4. Sound ratings

For each question, participants were presented with two vocalizations (A and B) produced by the same individual, which they could listen to by clicking on them as many times as they wished. They were then asked to compare their emotional content on the arousal or valence dimension. For the arousal questions, participants had to select one option among the three following possibilities: ‘A is HIGHER arousal than B’, ‘A is LOWER arousal than B’ or ‘Not sure’. For the valence questions, they had to choose between ‘A is POSITIVE (and B is negative)’, ‘A is NEGATIVE (and B is positive)’ or ‘Not sure’. Which of sound A or B was lower/higher arousal or negative/positive was randomized. To avoid preference for one answer over another because of its position on the page in the multiple-choice questions, the software randomly selected in which order the three answer options would appear on the page for each participant. However, to avoid confusion, the order of presentation of the options then remained the same throughout the survey for that participant.

Neither the species nor the context of production was revealed to the participants. Therefore, although they could very likely hear the difference between calls of horses and pigs for example, they could probably not differentiate calls of pigs and wild boars, or calls of domestic and Przewalski's horses. Above each question the concepts of arousal and valence were recapped, and participants were told that they could listen to each recording as many times as they liked.

In total, each participant was presented with a subset of four questions for each species: two for arousal and two for valence (2 arousal questions × 2 valence questions × 7 species = total 28 questions). These four questions were selected randomly by the survey software from the total number questions available for each species (see Survey stimuli for details). The order in which the questions from each species were presented was also randomized.

#### 2.2.5. Empathy score

After rating the sounds, the participants were given the option to complete a short questionnaire to assess their level of empathy. Empathy scoring was assessed using the interpersonal reactivity index (IRI) [44], in which participants are asked to mark their reaction to a series of questions, based on which their score in four dimensions of empathy can be calculated (empathic concern, fantasy, perspective taking and personal distress [44]). In total, 731 participants completed this questionnaire. Validated versions of this test were available in all the languages used in our survey (French [45], Spanish [46], Italian [47], Portuguese [48], German [49], Dutch [50] and Hebrew [51]).

### 2.3. Survey stimuli

For each species, we prepared a set of 4–10 questions (mean ± s.d. = 8.00 ± 2.38) for emotional arousal (i.e. 4–10 pairs of A and B sounds of higher and lower arousal) and 4–10 questions (mean ± s.d. = 9.00 ± 2.24) for emotional valence (i.e. 4–10 pairs of A and B sounds of opposite valence). Each valence question included one single vocalization associated with positive valence and the other with negative valence, both with a similar arousal level. Each arousal question included one vocalization associated with higher arousal and the other with lower arousal, both with the same valence. The two vocalizations in each question were produced by the same individual, were of the same call type (whinnies for horses and Przewalski's horses, grunts for pigs and wild boars, bleats for goats, moo for cow, meaningless speech sounds for humans) and had the same amplitude (all sounds were scaled to a relative absolute peak amplitude of 0.99 using Praat 5.3.41 [52]).

The non-human animal (hereafter ‘animal’) vocalizations used in our survey were individual contact calls recorded as part of previous studies on vocal expression of emotions (horses [21]; pigs [20]; goats [7]; cattle [24]; Przewalski's horses [22]; and wild boars [23]). They had been recorded in situations of known valence and arousal, and our acoustic analyses had revealed indicators of both emotional valence and arousal in all species. To summarize, the subjects had been recorded in situations *a priori* assumed to be associated with positive valence (i.e. with the pleasant-appetitive motivational system; e.g. short anticipation for food, affiliative interactions, social reunion) or with negative valence (i.e. with the unpleasant-defensive motivational system; e.g. food frustration, agonistic interactions, social separation and isolation). The emotional valence was then validated *a posteriori* using behavioural indicators described in the literature. The emotional arousal associated with the situations was assessed based on the heart rate of the animals for domestic species and on locomotion (good behavioural indicator of arousal, as assessed based on the domestic species results) for the wild species.

The human stimuli were actors' voices uttering meaningless strings of sounds to portray different emotions from the validated Geneva Multimodal Emotion Portrayal corpus [53]. To match the animal sounds and their subtle acoustic differences (based on human ear), for arousal questions, we chose sounds mimicking emotions that only slightly differed in arousal level but were of same valence: rage (hot anger) for higher arousal and fear for slightly lower arousal [54]. For valence questions, we chose sounds mimicking anger for negative valence and joy for positive valence (similar arousal) [55]. Human sounds were reduced to one pseudospeech sentence and were only slightly longer than animal sounds (duration (mean [range]): humans = 2.08 [1.00–4.94] s; animal sounds = 1.07 [0.19–4.17] s).

We analysed all prepared sound stimuli using a custom-based Praat script (adapted from [56,57]), which batch processed the vocalizations, analysed the listed parameters and exported those data for further evaluation. We extracted the following vocal parameters, which were known to be affected by emotions and could be measured in all species [9]: the duration (‘*Dur*’), the mean fundamental frequency (‘*f0*’), the spectral centre of gravity (‘*Q50%*’), and the variation in amplitude modulation (‘*AM*’) (see previous papers for more details [7,20,23]). Since human acted emotional vocalizations differ from spontaneous ones (both speech [58] and non-verbal vocalization [59]), by showing more pronounced expression of emotions, we used a similar approach to select the animal sounds for inclusion in the survey; we selected among our database, high-quality calls (i.e. with a low level of background noise) whose vocal parameters were representative of characteristic lower/higher arousal or negative/positive vocalizations for each species, based on our previous results [7,20–24]. This was done by sorting calls according to each main indicator of arousal or valence previously described for each species, and selecting the calls that were above average in all of these parameters, working from the most typical on. The extracted vocal parameters were also included in our analyses to test their effects on the participants’ rating of the stimuli.

To avoid pseudoreplication, for each species, we maximized the number of questions prepared (4–10 pairs of sounds for both arousal and valence for each species) and the number of individuals included in our selection of calls. In species for which we had less good quality calls, some calls were re-used to prepare arousal and valence questions (e.g. a low arousal negative call could be used both for an arousal question, paired with a high arousal negative call, and for a valence question, paired with a low arousal positive call). Overall, however, only 22 calls on a total of 216 calls were re-used to build two questions. In total, the number of individuals and calls used to prepare the survey was as follows: pigs = 14 individuals and 37 calls for 20 questions (10 arousal and 10 valence); horses = 11 individuals and 39 calls for 20 questions (10 arousal and 10 valence); goats = 9 individuals and 27 calls for 14 questions (4 arousal and 10 valence); cattle = 3 individuals and 28 calls for 16 questions (6 arousal and 10 valence); wild boars = 11 individuals and 39 calls for 20 questions (10 arousal and 10 valence); Przewalski's horses = 6 individuals and 19 calls for 11 questions (7 arousal and 4 valence); humans = 10 individuals and 27 pseudospeech sentences for 18 questions (9 arousal and 9 valence).

### 2.4. Statistical analysis

We used generalized linear mixed models (GLMMs) in R v.4.1.2 [60] to test whether the ratings of the participants were correct or not (their ‘scores’), and the factors influencing both these scores and how participants rated the sounds as lower/higher arousal or negative/positive valence (their ‘rating’). In these models, the answers rated by participants as ‘Not sure’ as well as blank answers were omitted. We tested different questions in separate models as follows. Since the outcome variable was always binary (0 or 1), we fitted all models with binomial family distribution and logit link function (GLMM, glmer function, package lme4 [61]).

#### 2.4.1. Ability to rate emotions across dimensions

First, we used a binomial test to compare the number of correct and incorrect responses for each dimension, as well as the number of ‘Not sure’ responses given between dimensions (all species combined and then by species; electronic supplementary material, table S2). Since binomial tests do not control for repeated measures of the same participants, we additionally used a GLMM to test the effect of the correct arousal (lower/higher) and the correct valence (negative/positive) on how participants rated the emotional arousal and valence of the sounds (as lower/higher arousal or negative/positive valence). An increase of the rating as higher arousal when the vocalization was indeed of higher arousal (or as positive when the vocalization was indeed positive for valence) would give another indication that participants can correctly rate the emotions of the vocalizations. This GLMM included how participants rated the sounds of all species (binary variable: lower arousal = 0, higher arousal = 1; negative valence = 0, positive valence = 1) as an outcome variable, and the correct arousal or the correct valence (lower/higher arousal or negative/positive valence), the species played (seven-level nominal variable: pigs, horses, goats, cattle, wild boars, Przewalski's horses or humans) as well as the interaction between these two factors as fixed factors, to find out if there was variation between species in the impact of the correct arousal or valence on the ratings, and hence on how well people could correctly rate arousal and valence (electronic supplementary material, table S3).

#### 2.4.2. Emotional dimensions

We tested if the emotional dimension (arousal or valence) affected participant ability to correctly rate the sounds of all species (including humans). This GLMM included the scores of the participants as an output variable (incorrect = 0, correct = 1), and the dimension (two-level nominal variable: arousal or valence), the species played (seven-level nominal variable: pigs, horses, goats, cattle, wild boars, Przewalski's horses or humans), as well as the interaction between these two factors as fixed factors (electronic supplementary material, table S4).

#### 2.4.3. Demography

We tested if any of the demographic factors collected in our survey influenced participant ability to correctly rate the sounds of other species (excluding humans). This was done by running a GLMM including the scores of the participants as an output variable, and the following fixed factors: the age of the respondents (five-level ordinal variable: < 20 years old = 0, 20–29 years old = 1, 30–39 years old = 2, 40–49 years old = 3, ≥ 50 years old = 4; excluding answers ‘I prefer not to say’), whether they had children or not (binary variable: yes = 1 or no = 0), their education level (two-level nominal variable: bachelor's degree or not; electronic supplementary material, table S5) and their gender (two-level nominal variable: male or female; excluding answers ‘I prefer not to say’).

#### 2.4.4. Empathy

We tested the impact of participants' empathy on their ability to correctly rate the sounds of other species (excluding humans), using a GLMM including the scores of the participants as an output variable, and the four subscales of the IRI (affective empathy: empathic concern and personal distress; cognitive empathy: perspective taking, fantasy) as fixed factors (all discrete numerical variables; electronic supplementary material, table S6).

#### 2.4.5. Familiarity

We tested if participant familiarity with the species impacted on their ability to correctly rate the sounds of other species (excluding humans) using a GLMM including the scores of the participants as an output variable and the following fixed factors: if participants had contact with each species included in the survey, and if so, how regularly this contact occurred (measured on a scale from never to daily; seven-level ordinal variable: never = 0; less than once a month = 1; once a month = 2; 2–3 times a month = 3; once a week = 4; 2–6 times a week = 5; daily = 6), whether they had received an education on a subject related to animals (binary variable: yes = 1 or no = 0), and whether their work was involving animals (e.g. veterinarian, ethologist, animal caretaker, farmer; binary variable: yes = 1 or no = 0; electronic supplementary material, table S7).

#### 2.4.6. Domestication

We tested the effect of the status of each species as domesticated or not on the ability of participants to correctly rate their sounds (excluding humans) using a GLMM, which included participant scores as an output variable, and the species domestication (binary variable: ‘0’ for Przewalski's horses and wild boars and ‘1’ for horses, pigs, goats and cattle) as a fixed factor. Since it was likely that participants would be more familiar with domestic than wild species, resulting in familiarity being a confounding factor, we also included the frequency of contact with each species as a control factor (seven-level ordinal variable, as described above; electronic supplementary material, table S8).

#### 2.4.7. Vocal parameters

We investigated two questions related to the vocal parameters of the sounds of other species (excluding humans) that were played: (i) whether the difference in the vocal parameters of the two sounds presented in each question influenced the ability of participants to correctly rate them (scores) and (ii) whether the parameters of the sounds influenced the participants' ratings of the sounds as lower/higher arousal or negative/positive valence (i.e. independently from whether ratings were correct or not). To investigate the first question, we ran a GLMM for each acoustic parameter measured, and for each of the emotional dimensions, including the scores of the participants as an output variable, and the difference in the parameter between the two vocalizations (A and B) presented in the same question as a fixed factor (continuous variable), along with the species (six-level nominal variable: pigs, horses, goats, cattle, wild boars or Przewalski's horses) as a second fixed factor and the interaction term between these two factors (electronic supplementary material, tables S9–S13). To investigate the second question, we ran a GLMM for each acoustic parameter measured, and for each of the emotional dimensions, including the ratings of the participants (as lower/higher arousal or negative/positive valence) as an outcome variable, and the value of the parameter measured in the first vocalization presented in each question (A) as a fixed factor (continuous variable), along with the species (six-level nominal variable: pigs, horses, goats, cattle, wild boars or Przewalski's horses) as a second fixed factor and the interaction term between these two factors (electronic supplementary material, tables S14–S18).

All models including only one valence dimension (arousal or valence questions only) had the following random structure: the species played (since each participant was tested with two arousal and two valence question per species) nested within the coding number of the participants (since each participant was tested with 14 questions per dimension), itself nested within their country of origin (since we collected data from up to 120 participants per country of origin). In addition, we included the species played (since each participant was tested with two arousal and two valence question per species) and the participants’ country of residence as separated crossed random effects (since we collected data from up to 118 participants per country of residence). For models conducted on each species separately, the random factor ‘species’ was omitted. For the models that included both valence and arousal questions, we added the dimension (valence or arousal; since each participant was tested with two arousal and two valence questions per species) in the nested random effect (dimension within species, within coding number, within participant's country of origin). This allowed us to control for dependency between data related to all of these factors and to account for the hierarchical design of the experiment accurately to prevent pseudoreplication (e.g. [62]; see electronic supplementary material, tables S3–S18, for the extract structure of each model).

To assess the effects of factors included in the above-described models, we used a Bayesian approach and compared all possible models based on the Bayes information criterion (BIC) using the function dredge (MuMIn library [62]), after verifying the absence of multi-collinearity between our factors (vif function car library [63]). A factor was estimated to have a strong effect if the difference in BIC between the best model and the model containing the factor (ΔBIC) was of 0 ≤ ΔBIC < 2, a medium effect if 2 ≤ ΔBIC < 4, a weak effect if 4 ≤ ΔBIC < 7 and no effect if ΔBIC ≥ 7 [64]. When a factor appeared in several models showing some support (ΔBIC < 7), we extracted its weighted parameter estimates (*β*) and standard errors (s.e.) using the function model.avg (MuMIn library). We used BIC instead of the now more commonly used AIC because BIC penalizes the number of explanatory factors included in the model more strongly than AIC (thus selecting a smaller number of models and preventing overfitting more efficiently) and allows a more parsimonious approach [65]. When the interaction term with the species was included within the best model(s), we ran further models on each species separately, using a similar approach (BIC).

Finally, we calculated the intraclass correlation coefficient (ICC) between the example question and when this same question was repeated later on. This showed moderate agreement (ICC = 0.55 95% CI [0.52, 0.59]), despite these ICCs being calculated based on the first question presented (example question).

## 3. Results

### 3.1. Ability to rate emotions: are humans able to correctly rate the emotional arousal and valence of the sounds of all species?

Overall, across species, people were able to rate both arousal (54.1% correct) and valence (55.3% correct) of the vocalizations slightly above chance levels (binomial test: *p* < 0.001 for both dimensions; figure 1*b,c*). Further binomial tests per species and dimension showed that the arousal of the vocalizations was correctly rated above chance level for pigs, horses, goats and humans (binomial test: *p* < 0.001 for all), but not for cattle, wild boars and Przewalski's horses (*p* ≥ 0.062 for all; figure 1*b*; electronic supplementary material, table S2). Respondents were able to correctly rate the valence of the vocalizations above chance levels for pigs, horses, goats, wild boars and humans (binomial test: *p* < 0.001 for all), while they rated the valence of cattle and Przewalski's horses significantly below chance level (*p* ≤ 0.020 for both; figure 1*c*; electronic supplementary material, table S2). Across species, the number of ‘Not sure’ answers did not vary significantly between arousal and valence questions (binomial test: *p* = 0.070; electronic supplementary material, table S2). When running tests at the species level, however, the number of ‘Not sure’ answers differed between arousal and valence questions for pigs, horses, goats, wild boars and humans (binomial test: *p* ≤ 0.027 for all; electronic supplementary material, table S2), but not for cattle, wild boars and Przewalski's horses (*p* ≥ 0.113 for all; electronic supplementary material, table S2).

A GLMM testing the effect of the correct arousal or valence and the species played (including answers to human-related questions) as well as their interaction on participant ratings (as lower or higher arousal for arousal questions; or negative or positive valence for valence questions) revealed that the best model selected based on the BIC for arousal questions was the model including the correct arousal only (probability to be the best model (*ω*i) = 100%; electronic supplementary material, table S3). This suggests that people ratings of arousal did not differ much between species (overall slope estimate for the effect of correct arousal on rated arousal = 0.34; figure 1*b*). By contrast, for valence questions, the best model was the model with the correct valence, the species and the interaction between these two factors (*ω*i = 100%; electronic supplementary material, table S3), indicating that ratings of valence varied between species. Further models conducted on each species separately revealed that the correct valence was included in the best model for all species (*ω*i = 100% for all) but cattle (electronic supplementary material, table S3). For cattle, the best model was the model with the intercept only, but the model with the correct valence had a BIC that differed from the best model by less than 2 (*ω*i, ΔBIC: cattle = 29.3%, 1.8), indicating that the correct valence had an effect on how participants rated the sounds of this species. Slope estimates indicated a positive relationship between correct valence and rated valence for pigs (0.64), horses (1.14), goats (0.57), wild boars (0.84) and humans (1.50), while a negative relationship was observed for cattle (−0.24) and Przewalski's horses (−1.74) (electronic supplementary material, table S3). This suggests that people correctly rate the valence of pigs, horses, goats, wild boars and human sounds, while they incorrectly rate the valence of cattle and Przewalski's horses (for percentages of correct recognition, see figure 1*c*; electronic supplementary material, table S2). Correct rating of arousal is thus less dependent of the species played than correct rating of valence, for which some species (cattle and Przewalski's horses) are incorrectly rated (i.e. positive sounds are rated as negative, and vice versa).

### 3.2. Emotional dimensions: is arousal or valence easier to rate across species?

A GLMM with the scores of participants as an output variable, the emotional dimensions (arousal or valence) and the species played (including answers to human-related questions) as well as their interaction revealed that the best model was the model containing all three terms (dimension, species and their interactions; *ω*i, ΔBIC = 100%, 0.0) (electronic supplementary material, table S4). Further models conducted on each dimension separately showed that for arousal, both the model with species (*ω*i, ΔBIC = 73.7%, 0.0) and the null model (26.6%, 2.0) were competitive (ΔBIC < 4) (electronic supplementary material, table S4). By contrast, for valence, the model with species (*ω*i, ΔBIC = 100%, 0.0) had much more support than the null model (0.0%, 491.6; electronic supplementary material, table S4). This suggests, once more, that correct rating of valence is much more species dependent than correct rating of arousal.

### 3.3. Demography: does participant demography influence their scores?

**Figure 2.**
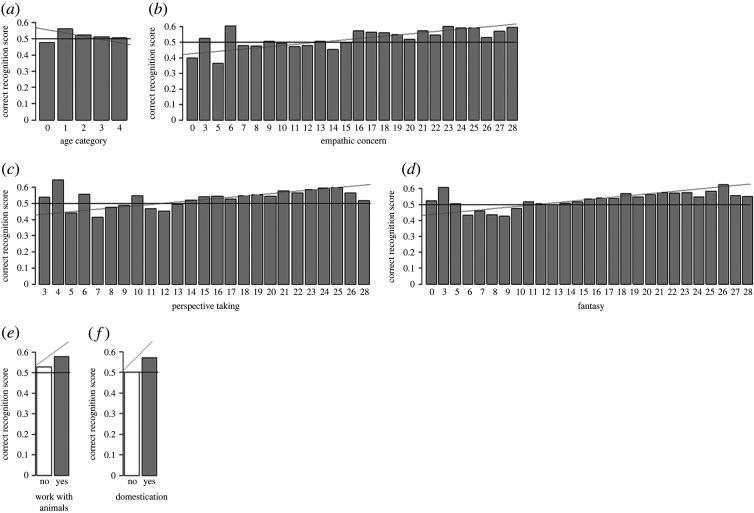
Correct recognition score as a function of (*a*) age category, (*b*) empathic concern, (*c*) perspective taking, (*d*) fantasy, (*e*) work with animals and (*f*) domestication; bar plots: the grey lines show the model slope estimate and the black lines the chance level.

Based on a GLMM with the scores of the participants as an output variable (excluding answers to human-related questions) and the following factors: the age of the respondents (categories 0–4), whether they had children or not, whether they had completed a bachelor's degree or not, and their gender (male or female), we found that the model with the respondent age only (*ω*i, ΔBIC = 56.0%, 0.0) and the null model (39.5%, 0.7) were highly competitive (ΔBIC ≤ 2) (electronic supplementary material, table S5). This suggests that participants in older age categories tended to be less correct in their ratings than younger ones (slope estimates = −0.06) (electronic supplementary material, table S5), with the exception of the youngest age category (less than 20 years old), who also had low scores (figure 2*a*). Therefore, with the exception of age that had a negative impact on participant scores, none of the demographic factors that we tested affected the correct ratings of expression of emotions of species other than humans by our participants.

### 3.4. Empathy: does participant empathy towards other humans influence their scores?

A GLMM with the scores of the participants as an output variable (excluding answers to human-related questions) and the four subscales of the IRI (affective empathy: empathic concern and personal distress; cognitive empathy: perspective taking, fantasy) as factors revealed that four different models had some support (ΔBIC < 7) (electronic supplementary material, table S6). These were a model with only fantasy (*ω*i, ΔBIC = 81.7%, 0.0), a model with only empathic concern (7.5%, 4.8), a model with fantasy and empathic concern (4.9%, 5.6), and a model with fantasy and perspective taking (3.7%; 6.2) (electronic supplementary material, table S6). This suggests that empathic concern (slope estimate ± s.e. = 0.018 ± 0.007; figure 2*b*), perspective taking (0.011 ± 0.006; figure 2*c*) and fantasy (0.021 ± 0.005; figure 2*d*) all have a positive effect on correct ratings of vocal expression of emotions. Thus, several aspects of both affective and cognitive empathy towards conspecifics improve our ability to recognize vocal expression of emotions across species.

### 3.5. Familiarity: does participant familiarity with each species influence their scores?

A GLMM with the scores of participants as an output variable (excluding answers to human-related questions) and the following factors: if participants had contact with each species included in the survey, and if so, how regularly this contact occurred (measured on a scale from never (0) to daily (6)), whether they had received an education on a subject related to animals or not, and whether their work involved animals or not, revealed that the best model was the null model (intercept only; *ω*i = 87.9%) (electronic supplementary material, table S7). However, the second model also had some support (ΔBIC < 7) and included only work with animals (*ω*i, ΔBIC = 7.9%, 4.8) (electronic supplementary material, table S7). This suggests that people working with animals are, to a limited extent, better at recognizing vocal expression of emotions in animals than those who do not (slope estimate = 0.20) (electronic supplementary material, table S7; figure 2*e*). Thus, the amount of exposure to other species' vocalizations can improve emotion perception, suggesting a role of experience-driven cognitive processes in emotion recognition.

### 3.6. Domestication: does domestication of the species influence participant scores?

A GLMM using participant scores as correct or not as an output variable (excluding answers to human-related questions), species domestication (‘No’ = 0, for Przewalski's horses and wild boars and ‘Yes’ = 1 for horses, pigs, goats and cattle) as a fixed factor, and the frequency of contact with each species as a control factor showed that the best model was the null model (intercept only; *ω*i = 91.6%) (electronic supplementary material, table S8). However, the model containing only domestication also had some support (*ω*i, ΔBIC = 6.3%, 5.4) (electronic supplementary material, table S8). This indicates that whether a species is domesticated or not plays a role, as participants were better able at scoring emotions in vocalizations of domestic than wild species (slope estimate = 0.32; figure 2*f*).

### 3.7. Vocal parameters: does the difference in acoustic parameters between the two sounds in each question influence participant scores?

We first ran one GLMM per acoustic parameter for each of the emotional dimensions, including participant scores as an output variable, and the difference in this parameter between the two vocalizations presented in a given question as a fixed factor, along with the species as a second fixed factor and the interaction term between these two factors. For arousal, the model selection revealed that the differences in *f0* (*ω*i, ΔBIC = 1.7%, 8.1) and *AM* (1.7%, 8.1) between the two sounds presented in each question had no effect on the participants’ scores (electronic supplementary material, table S9). However, the model including only the difference in *Dur* and in *Q50%* was rated as the second best model (*ω*i, ΔBIC: *Dur* = 3.9%, 6.4; *Q50%* = 6.8%, 5.3), behind the null model (*ω*i: *Dur* = 96.1%; *Q50%* = 93.2%) (electronic supplementary material, table S9). This suggests that a smaller difference in *Dur* (slope estimate = −0.04) and a larger difference in *Q50%* (0.06) between the two sounds slightly improve our ability to correctly rate emotional arousal across species (electronic supplementary material, table S9).

For valence, the models including the difference in the parameters between the two vocalizations, the species and the interaction between the two factors were rated as the best models for all four parameters (*ω*i: *Dur* = 100%; *f0* = 99.8%; *Q50%* = 70.0%; *AM* = 100%; electronic supplementary material, tables S10–S13). This indicates that either a larger or smaller difference in these four parameters between the two presented sounds improved participant scores, depending on the species. The relationship (slope estimate) between correct ratings and difference in *Dur* between the two sounds ranged between −0.94 for Przewalski's horses and 0.58 for pigs (electronic supplementary material, table S10). For *f0*, it ranged between −0.46 for cattle and 0.12 for horses (electronic supplementary material, table S11). For *Q50%*, it ranged between −0.17 for wild boars and 0.73 for Przewalski's horses (electronic supplementary material, table S12). Finally, for *AM*, it ranged between −0.43 for cattle and 0.56 for pigs (electronic supplementary material, table S13). For *Q50%*, the second best model was the model including only the difference in this parameter between the two sounds (*ω*i, ΔBIC = 29.8%, 1.7), suggesting that a larger difference in *Q50%* (slope estimate = 0.41) improved participants’ ability to correctly rate emotional valence across species (electronic supplementary material, table S12).

Therefore, the effect of the difference in parameters between the two sounds presented in each question was similar across species for arousal: smaller differences in *Dur* and larger differences in *Q50%* improved the scores of the participants across species, while differences in *f0* and *AM* had no effect. Contrastingly, for valence—with the exception of *Q50%* for which there was additional evidence that a larger difference between the two sounds improved the scores across species—the effect of the difference between the two sounds in all parameters varied from negative to positive effects depending on the species.

### 3.8. Vocal parameters: do the acoustic parameters of sounds influence their ratings by participants as lower/higher arousal or negative/positive valence?

We then ran one GLMM per acoustic parameter for each emotional dimension, including how participants rated the sounds (ratings as lower/higher arousal or negative/positive valence) as an output variable, and the value of the parameter measured in the first vocalization presented in the corresponding question as a fixed factor, along with the species as a second fixed factor and the interaction term between these two factors. For arousal, the model selection revealed that the best model for *Dur*, *Q50%* and *AM* was the model with only the value of these parameters (*ω*i: *Dur* = 71.3%; *Q50%* = 97.9%; *AM* = 100%), although for *Dur*, the null model was competitive (*ω*i, ΔBIC: *Dur* = 28.6%, 1.8) (electronic supplementary material, table S14). For *f0*, the model including only the value of this parameter was rated as the second best model (*ω*i, ΔBIC: 5.1%, 5.8), behind the null model (*ω*i: *Dur* = 94.9%), suggesting a moderate effect of this factor (electronic supplementary material, table S14). This suggests that a longer *Dur* (slope estimate = 0.12), a higher *f0* (0.06), a higher *Q50%* (0.15) and a smaller *AM* (−0.19) lead to higher arousal ratings across species (electronic supplementary material, table S14).

For valence, the best models for *Dur*, *f0* and *AM* were those including the parameter values, the species and the interaction between these two terms (*ω*i = 100% for all), indicating differences between species in the effect of these parameters on valence ratings (electronic supplementary material, tables S15, S16 and S18). For instance, longer *Dur* was rated as more positive in Przewalski's horses (slope estimate = 1.11), but more negative in domestic horses (−0.44) (electronic supplementary material, table S15). Higher *f0* was rated as more positive in Przewalski's horses (slope estimate = 0.91), but more negative in cattle (−0.29) (electronic supplementary material, table S16). Finally, larger *AM* was rated as more positive in Przewalski's horses (slope estimate = 0.90), but more negative in cattle (−0.16) (electronic supplementary material, table S18). By contrast, for *Q50%*, the best model was the model including only the value of this parameter (*ω*i = 100%), suggesting that a higher *Q50%* (slope estimate = −0.24) leads to more negative valence ratings across species (electronic supplementary material, table S17).

Overall, the effect of the acoustic parameters on ratings of arousal were similar across species, with longer *Dur*, higher *f0* and *Q50%*, and lower *AM*, leading participants to perceive the sounds as higher in arousal. On the other hand, for valence, as predicted, this effect varied between species for all parameters measured, except *Q50%*, with some parameters resulting in ratings of sounds as more positive in some species and more negative in others.

## 4. Discussion

In order to decipher the evolution of emotion expression, we investigated if humans are able to perceive subtle changes in the acoustic structure of the vocalizations of domestic ungulates and their closely related species, as well as the factors affecting this perception of emotions, namely the emotional dimensions (arousal or valence), demographic factors, empathy, familiarity with the species or with animals, domestication and the acoustic features of the sounds. Overall, our results show that participants could correctly rate the emotional arousal and valence of the human sounds and of several of the domesticated and wild ungulates used in this survey, even though the correct ratings (scores) obtained throughout were rather low (55–68% for those above chance level), possibly due to the short duration of the sounds used (humans, 1.00–4.94 s; ungulates, 0.19–4.17 s). Scores for arousal were above chance level for human sounds and for three of the six of ungulates we played (pigs, horses and goats), with a relatively similar performance across species (scores above chance level: 55–59%). On the other hand, scores for valence differed widely between species, with some correctly rated well above chance levels (56–68%: humans, horses, goats, pigs and wild boars), and others being incorrectly rated (below chance levels; 33–47%: cattle and Przewalski's horses). Along those lines, we found that the effect of the difference in vocal parameters between the two sounds played in each arousal question on participant scores was similar across species, while for valence questions, large difference existed between species. Finally, similar conclusions could be reached when looking at the effect of the parameters of the sounds presented (instead of the difference between the two sounds) on how people rated them; these effects were similar across species for arousal ratings, while they differed a lot between species for valence questions. Combined, these findings are in line with previous claims (e.g. [9,10]), suggesting the existence of a shared emotional arousal system across mammalian species. It could thus be that the expression of emotional arousal has been conserved throughout evolution, while the expression of emotional valence, with the exception of few parameters (e.g. duration), has not.

Our correct ratings of human sounds were in general rather low, particularly for arousal (i.e. only slightly above chance levels, 55%, compared to 68% for valence). There could be two main explanations for this low success. First, to match the duration of the animal sounds, we had cut the sounds down to only one pseudospeech sentence (1.00–4.94 s). Second, to match the difficulty of the task of rating human sounds versus animal sounds, for arousal, we chose actors’ portrayals of hot anger as higher arousal versus fear as ‘lower’ arousal. Although these two emotions are predicted to slightly differ in arousal (e.g. Plutchik's wheel of emotions [54]; physiological studies show that anger triggers slightly higher heart rate than fear [66]), their arousal is very close (both high [55]). These two emotions and their portrayals might have not differed enough to be rated easily by participants, unlike, for example, sadness and anger, which were correctly classified 95% of the time in a similar task in Filippi *et al*. [10]. Concerning the animal sounds, their scores ranged from 49% to 59% for arousal, which is also not very high compared to previous studies (e.g. 60% to 94% correct ratings of arousal across eight species [10]), while our scores for valence (33% to 68%) closely match those found for instance for ratings of silver fox vocalizations (30% to 65% [43]) and horse whinnies (64.4% in [67] versus 64% in our survey). On the one hand, we selected for our survey calls that were the most representative in their parameters of the emotions used (lower/higher arousal and negative/positive valence) based on our previous studies, which might have made the task slightly easier. On the other hand, participants had to compare single calls of the same type (all contact calls), which were sometimes very short (as low as 0.19 s). We thus believe that the difficulty of the task might explain the rather low correct ratings that we obtained throughout. The particularly low scores obtained for Przewalski's horses (33%) could be due to participants relying on their knowledge of domestic horses' expression of valence, which differs substantially from Przewalski's horses [22]. In addition, horse was the non-human species our participants were the most familiar with (mean ± s.d. contact species scale = 1.09 ± 2.01), while Przewalski's horse was the least familiar (contact species scale = 0.04 ± 0.31). However, the low scores obtained for cattle arousal (47%) cannot be explained by familiarity (contact species scale = 0.62 ± 1.45) nor domestication, and should be investigated further.

In contrast with some previous studies (e.g. [28,67]), we did not find an effect of gender on correct ratings. In opposition to our predictions, we also did not find any effect of having children or not or education. However, our analyses revealed an effect of age that reflects previous findings, with a decrease in correct ratings with age, except for the youngest age class (less than 20 years old), which had low ratings. It could thus be that correct recognition of ungulate emotions improves with age in young participants (e.g. children compared to adults), as has beenshown for recognition of emotions in stump-tailed macaque vocalizations [36] and dog vocalizations [26], and that it then decreases in older age groups, in the same way as for certain human emotions across modalities [35], including the auditory modality [68].

We also found an effect of empathy on recognition of animal vocal expression of emotions, similarly as found for cat meows produced in isolation [69]. Indeed, three of the four factors measured by the IRI questionnaire (perspective taking, empathic concern and fantasy), which encompass both emotional and cognitive empathy, had a positive effect on correct ratings of emotions. People rated as more empathetic towards others could thus have an advantage for understanding the emotions of both domestic and wild species.

In agreement with some previous studies (e.g. [16,17,33]), we found that familiarity, in terms of work related to animals, improved the ability of participants to correctly rate vocal expressions of emotions. Domestication is suggested to have played a role in human–animal communication of emotions; furthermore, it seems to have enhanced dog–human communication [8]. Our results do support this effect of domestication for ungulates as well, as the emotion expressions of domesticated species were more correctly recognized than wild ones.

Our results show that the acoustic structure of the sounds affects correct ratings of emotional expression. Overall, across species, smaller differences in duration between the two sounds presented in each question improved arousal scores. In addition, larger differences in the spectral centre of gravity (*Q50%*) between the two sounds improved scores for both arousal and valence. *Q50%* commonly increases with arousal across species [5]. Larger differences in this parameter might thus have made the difference between the lower and higher arousal call more obvious. By contrast, this parameter is not clearly related to valence, as its effect depends on the species, with some having higher *Q50%* in negative than positive situations [23], and others showing the opposite effect [70], unlike *f0*, which is often lower in positive compared to negative situations across species [5]. It is therefore not clear why larger differences in *Q50%* improved valence scoring in our study. Duration, on the other hand, is often shorter in positive compared to negative situations [5] and is thus more related to valence than arousal. This could explain why smaller differences in duration between the two sounds improved arousal scoring, by allowing participants to focus their attention on other, more relevant vocal parameters for this emotional dimension (e.g. *Q50%*).

Concerning the effect of the parameters on emotion perception, we found that sounds with longer durations, higher *f0* and *Q50%* and lower *AM* led participants to rate the sounds as higher in arousal. This is partly consistent with changes occurring across species (for *f0* and *Q50%*) [5], and consistent with what has been found in other similar studies of human perception of both human and animal sounds (e.g. [10,17,25,52]). The effect of parameters on valence perception varied between species, except for *Q50%,* which induced more negative valence ratings across species when higher. Overall, as we predicted, the parameters facilitating arousal recognition (difference between the two sounds) and affecting arousal perception were similar across species, while those enabling valence recognition and affecting valence perception differed between species.

## 5. Conclusion

Our study adds to the evidence suggesting that expression of emotional arousal has been conserved throughout evolution, while valence expression might be more species specific. In addition, we show that our ability to judge animal emotions depends on our age, capacity for empathy, our familiarity with animals and whether they have been domesticated or not. By contrast, this ability seemingly does not depend on demographic factors such as gender, having children or level of education.

## Data Availability

The data that support this study are provided as an electronic supplementary material file (DataFile S1) [71].
